# Metabolomics Analysis of Different Tissues of *Lonicera japonica* Thunb. Based on Liquid Chromatography with Mass Spectrometry

**DOI:** 10.3390/metabo13020186

**Published:** 2023-01-27

**Authors:** Yan Wang, Lili Li, Wenhua Ji, Shuang Liu, Jiali Fan, Heng Lu, Xiao Wang

**Affiliations:** 1 Key Laboratory for Applied Technology of Sophisticated Analytical Instruments of Shandong Province, Shandong Analysis and Test Center, Qilu University of Technology, Shandong Academy of Sciences, Jinan 250014, China; 2 College of Traditional Chinese Medicine, Yunnan University of Chinese Medicine, Kunming 650500, China; 3 School of Pharmaceutical Sciences, Qilu University of Technology, Shandong Academy of Sciences, Jinan 250014, China

**Keywords:** *Lonicera japonica* Thunb., metabolomics analysis, different tissues, liquid chromatography with mass spectrometry

## Abstract

*Lonicera japonica* Thunb. (LJT) has been widely used as medicines or food additives in Asian countries for thousands of years. The flower buds are often medicinally used, and the other tissues are ignored. However, flowers, leaves and stems have also been reported to have antimicrobial, anti-inflammatory and antioxidant effects. In the current study, un-targeted metabolomics analysis was performed to investigate the metabolic difference among different tissues (flowers, flower buds, stems and leaves) of LJT based on liquid chromatography with mass spectrometry. A total of 171 metabolites were identified, including 28 flavonoids, 35 phenolic acids, 43 iridoids, 9 amino acids, 6 nucleotides, 16 fatty acids, 22 lipids and 12 others. Four new secondary metabolites were discovered. Some flavonoids and iridoids were not detected in leaves and stems. Principal component analysis showed significant differences among four different tissues. Some 27, 81, 113 differential metabolites were found between flowers/flower buds, leaves/flower buds, stems/flower buds, respectively. Primary metabolites showed a higher content in the flowers and flower buds. For the flavonoids, flavones were mainly accumulated in the leaves, flavonols were mainly accumulated in the flower buds, and acylated flavonol glucosides were mainly accumulated in the flowers. Most phenolic acids showed a higher content in the flowers or flower buds, while phenolic acid-glucosides showed significantly higher content in the flower buds. The most abundant iridoids in the LJT also showed a higher content in the flowers and flower buds. These results can provide new insights into the understanding of the metabolites changes in different tissues, and lay a theoretical foundation for the comprehensive utilization of LJT.

## 1. Introduction

*Lonicera japonica* Thunb. (LJT), belonging to the Caprifoliaceae family, has been used in traditional Chinese medicine or as a food additive for thousands of years [1]. It is widely cultivated in China as well as in other Asian countries, such as Japan and Korea [2]. *Lonicera japonica* Thunb. has various medicinal effects, including anti-inflammatory, antiviral, antibacterial, antioxidant, hepatoprotective and anti-tumor, etc. [3]. The metabolites are directly related to biological functions. *Lonicera japonica* Thunb. contains many medicinal components such as flavonoids, phenolic acids, and iridoids [4]. Flavonoids are found to inhibit inflammatory cytokines and mediators [5]. Phenolic acids, especially caffeoylquinic acids and caffeic acid, show significantly antiviral and antioxidant activities [6]. Iridoids are found to have anti-inflammatory, antiviral, anticancer and analgesic activities [7].

The metabolites are structurally complex in the plant, especially secondary metabolites. Thousands of metabolites can be detected in plant tissues using mass spectrometry. Metabolomics is a research discipline that integrates the capabilities of several types of research including analytical chemistry, statistics and biochemistry [8]. Metabolomics analysis involves the detection and quantification of metabolites with mass spectrometry or nuclear magnetic resonance, and integrates the resulting data with multivariate statistical techniques [9,10]. With the development of liquid chromatography with mass spectrometry (LC-MS), metabolomics has been widely used in plant research [11]. There are abundant chemical components among different medicinal tissues. More organic acids and lipids have been found in the seeds than leaves and bark of *Eucommia ulmoides* Oliver with untargeted metabolomics [12]. A total of 62 components were selected as potential biomarkers to distinguish leaves, peels, flowers, pulps, peels and seeds for *Clausena lansium (Lour.)* Skeels [13]. Differential primary metabolites were significantly lower in the shoots than the roots, while secondary metabolites were higher in the shoots than in roots for *Salsola collina* Pall [14].

The flower buds of LJT have been medicinally used, while the flowers, leaves and stems have often been neglected, resulting in insufficient utilization. Recently, increasing amounts of research has focused on the chemical components of other tissues of LJT. Medicinal components have also been characterized. In the flowers, neochlorogenic acid, chlorogenic acid, cryptochlorogenic acid, 4,5-*O*-caffeoylquinic acid, sweroside and secoxyloganin have been found [15]. In the leaves, luteolin, rutin, luteoloside, lonicerin, isochlorogenic acid C, isochlorogenic acid B, isochlorogenic acid A and chlorogenic acid have been found [16]. In the stems, 18 medicinal substances such as neochlorogenic acid, chlorogenic acid, loganin, secoxyloganin, ochnaflavone, sweroside have been identified [17]. Previous studies have usually focused on several components in the individual tissues. Moreover, studies on dynamic primary and secondary metabolite changes among different tissues of LJT have been few. In the current study, qualitative and semi-quantitative analyses were performed on the flowers, flower buds, stems and leaves of LJT. The metabolomic profiling of the four different tissues was investigated, and different metabolites were identified. The metabolomic differences of LJT were analyzed and differential metabolites were filtered. The variations and regulation of metabolites among different tissues were investigated.

## 2. Materials and Methods

### 2.1. Chemicals and Materials

Acetonitrile and methanol (HPLC grade) were purchased from Merck (Darmstadt, Germany). Ultrapure water was made using a Direct-Q 8 UV-R water purification system (Millipore, Billerica, MA, USA). Standard components of chlorogenic acid, cryptochlorogenic acid, neochlorogenic acid, isochlorogenic acid A, isochlorogenic acid B, isochlorogenic acid C, 3-*O*-feruloylquinic acid, 5-*O*-feruloylquinic acid, cinnamic acid, caffeic acid, ferulic acid, coumaric acid, sinapic acid, luteolin, apigenin, luteoloside, rutin, apigenin-7-*O*-glucoside, isorhamnetin-3-*O*-glucoside, quercetin-3-*O*-glucoside, loganin, vogeloside were obtained from Shanghai Yuanye Biotechnology Co. Ltd. (Shanghai, China).

### 2.2. Plant Materials

**Figure 1:**
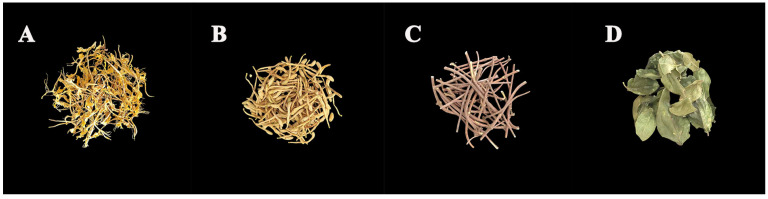
Phenotypes of different tissues of LJT. (**A**) flowers. (**B**) flower buds. (**C**) stems. (**D**) leaves.

Fresh LJT samples were collected in Linyi of Shandong Province, China. Four different tissues samples were collected separately from five plants: flowers (F), flower buds (FB), stems (S) and leaves (L) (Figure 1). Samples were immediately quenched in liquid nitrogen after collection. There were three biological replicates. The LJT samples were identified by Professor Wang Xiao at Shandong Analysis and Test Center.

### 2.3. Sample Preparation

The samples were freeze-dried, ground into powder, and passed through a 40-mesh sieve respectively. Quality control (QC) samples were obtained by mixing equal powder of each sample to be analyzed. The powder of 150 mg was accurately weighed into 5 mL Eppendorf tube. Subsequently, 3 mL precooled extraction solution of methanol/water (3:1, *v*/*v*) solution was added to the tube, and the tube was extracted in ice water with ultrasonic for 30 min. The solution was centrifuged at 12,000 rpm for 10 min, and then freeze-dried by a refrigerated centrifugal vacuum concentrator (Centrivap, Labconco, Kansas City, MO, USA). For LC-MS analysis, the samples were re-dissolved in 1 mL solution of 10% methanol/water (*v*/*v*) solution, and filtered through 0.22 μm membrane filters.

### 2.4. LC-MS Analysis

Analysis was performed with an UHPLC system (H-Class, Waters, Milford, MA, USA) combined with a Q-TOF mass spectrometer equipped with an ESI interface (Impact II, Bruker, Germany). The UHPLC analyses were performed on an Agilent ZORBAX SB C18 column (2.1 × 100 mm, 1.8 μm, Agilent, Palo Alto, CA, USA). The column oven was maintained at 40 °C and the flow rate was 0.3 mL/min. The temperature of the injection chamber was controlled at 4 °C. The mobile phases contained A (0.1% formic acid in water, *v*/*v*) and B (acetonitrile). The mobile phase B linearly increased from 5% to 25% during the first 20 min, and then linearly increased 100% in 15 min and was kept for 5 min. The total run time was 40 min. The mass spectrometry experiment was performed with the ESI source. The mass range was from *m/z* 50 to 1200. The capillary voltage was 3500 V in the positive mode and 3000 V in the negative mode. The nebulizer pressure was 2.0 bar, the flow rate of dry gas was 8.0 L/min, and the drying gas temperature was 200 °C. The collision radio frequency (RF) was 750 Vpp, the prepulse storage was 8 μs, and the transfer time was 80 μs.

### 2.5. Data Analysis

The data deconvolution and peak alignment were performed using MS-DIAL [18], and the peak table with *m/z*, retention time, and area was obtained. Principal component analysis (PCA) and orthogonal partial least squares discrimination analysis (OPLS-DA) were conducted using SIMCA (version 14.0, Umetrics, Umeå, Sweden). Nonparametric test and hierarchical cluster analysis (HCA) were conducted using MultiExperiment Viewer (version 4.9, Dana-Farber Cancer Institute, Boston, MA, USA). Pathway mapping of identified metabolites was performed using the Kyoto Encyclopedia of Genes and Genomes (KEGG) database (http://www.genome.jp/kegg/, accessed on 21 August 2022).

## 3. Results and Discussion

### 3.1. Metabolites Identification in Different Tissues

The metabolites were identified based on molecular weight, retention time and characteristic fragment ions. Standards available were used for confirmation. A total of 171 metabolites were identified, of which 171were in the flowers, 169 in the flower buds, 142 in the leaves and 139 in the stems. The 171 metabolites include 28 flavonoids, 35 phenolic acids, 43 iridoids, 9 amino acids, 6 nucleotides, 16 fatty acids, 22 lipids and 12 others (Table 1 and Table S1). Among them, secondary metabolites of phenolic acids, flavonoids and iridoids have specific fragment rules.

#### 3.1.1. Identification of Flavonoids

Flavonoids have three rings (C6-C3-C6) as their basic skeleton (labeled as A, B, and C) [19]. According to structural differences, flavonoids can be divided into flavonols, flavones, isoflavones and other subclasses [20]. Flavonoids have been found to be glycosylated and acylated. Flavonoids mainly existed in the form of *O*-glycosides in the LJT. The *O*-glycosidic bond is easily broken, showing the fragment ions of aglycone. Subsequently, the aglycone ions would be cleaved to form a series of fragment ions including dehydration, Retro Diels-Alder (RDA) fragmentation, C-ring fragmentation [21]. The peak 3 presented [M−H]^−^ at 431.0976 and fragment ions at 269 [M−H−Glu]^−^, 251 [M−H−Glu−H_2_O]^−^, 223 [M−H−Glu−H_2_O−CO]^−^. It was identified as apigenin-7-*O*-glucoside (Table 1). The peak 5 presented [M−H]^−^ at 447.0924 and fragment ions at 285 [M−H−Glu]^−^, 241 [M−H−Glu−CO_2_]^−^, 151 [^1,3^A^−^], 133 [^1,3^B^−^]. It was identified as luteoloside. 18 flavonoid *O*-glycosides were identified from LJT, including quercetin-*O*-glucoside, isorhamnetin-*O*-glucoside, quercetin-*O*-rutinoside, luteolin-*O*-diglucoside, kaempferol-*O*-diglucoside, apigenin-*O*-neohesperidoside, etc. Some flavonoid glycosides are further acylated. Acylated flavonoids were found in the flavonol subclass. Three acylated flavonoid glycosides were identified for the first time from LJT, including kaempferol-acetylglucoside, quercetin-acetylglucoside and quercetin-malonylglucoside. Among the flavonoids, 28 were detected in flowers and flower buds, 25 in leaves and only 17 in stems. Apigenin-5-*O*-neohesperidoside, apigenin-7-*O*-glucoside, isorhamnetin-3-*O*-glucoside, isorhamnetin-*O*-rutinoside I, isorhamnetin-*O*-rutinoside II, luteolin-*O*-diglucoside, kaempferol-*O*-rutinoside III, diosmin, diosmetin, corymbosin and flavoyadorinin B were absent in the stems. Corymbosin and flavoyadorinin B and 5-hydroxyl-3′,4′,7-trimethoxy flavone were absent in the leaves.

#### 3.1.2. Identification of Phenolic Acids

Phenolic acids were found in free forms or conjugated with quinic acid (QA)/glucoside in LJT [22]. Five free phenolic acids were identified in LJT, including caffeic acid, ferulic acid, coumaric acid, sinapic acid and cinnamic acid. The MS/MS spectra showed characteristic peaks of losing free phenolic acid, quinic acid or glucose. A total of 35 phenolic acids were identified, of which 5 were free phenolic acids, 28 were bound phenolic acids and 2 were others.

The peaks (29, 30 and 31) both presented [M−H]^−^ at *m/z* 353.0869. Their MS/MS spectra showed characteristic ions at *m/z* 191 or 173 and *m/z* 179. The elution order was confirmed by comparing with standards, and peaks 29, 30 and 31 were finally deduced as neochlorogenic acid, chlorogenic acid, cryptochlorogenic acid (Table 1). The peaks (37, 38 and 39) both presented [M−H]^−^ at *m/z* 515.1186. Their MS/MS spectra showed characteristic ions at *m/z* 353 or 335, 191 and 179. The elution order was confirmed by comparing with standards, and peaks 37, 38, and 39 were finally deduced as isochlorogenic acid B, isochlorogenic acid A, isochlorogenic acid C. The peaks (40, 41 and 42) both presented [M−H]^−^ at *m/z* 529.1338. Their MS/MS spectra had characteristic peaks at *m/z* 353 or 335 and 367. Peaks 40, 41 and 42 were preliminarily assigned as feruloyl caffeoylquinic acid and its isomers. Phenolic acid glycosides are more hydrophilic. The peak (49) presented [M−H]^−^ at *m/z* 515.1397. Its elution time was 3.78 min, and its MS/MS spectra had a characteristic peak at *m/z* 353. The peak 49 was finally deduced as caffeoylquinic acid-glucoside. The peak (50) presented [M−H]^−^ at *m/z* 529.1555. Its elution time was 4.98 min, and its MS/MS spectra had characteristic peak at *m/z* 367. The peak 50 was finally deduced as feruloylquinic acid-glucoside. Some 29 bounded phenolic acids were identified from LJT, including 3-*O*-feruloylquinic acid, 3-*O*-p-coumaroylquinic acid, sinapoylquinic acid, p-coumaroyl caffeoylquinic acid, caffeoyl sinapoylquinic acid, feruloylquinic acid-glucoside, sinapoylquinic acid-glucoside etc. In addition, sinapoylquinic acid-glucoside was identified for the first time from LJT. All these phenolic acids were detected in the flowers, flowers buds, leaves and stems.

#### 3.1.3. Identification of Iridoids

The iridoids in LJT were mainly in the form of glycosides. Iridoids exhibit characteristic fragmentations of loss of glucose, H_2_O, CO, and RDA cleavage [23]. The peaks (69, 70 and 71) both presented [M−H]^−^ at 389.1081. In the MS/MS spectrum, they showed fragment ions at *m/z* 209, 165, 121. The peaks 69, 70 and 71 were tentatively characterized as secologanoside and its isomers (Table 1). The peaks (80 and 81) both presented [M−H]^−^ at 375.1288. In the MS/MS spectrum, they showed fragment ions at *m/z* 213, 169, 151. The peaks 80 and 81 were tentatively characterized as 8-epi-loganic acid and 7-epi-loganic acid. The peaks 74 and 75 both presented [M−H]^−^ at 757.2550. In the MS/MS spectrum, they produced fragment ions at *m/z* 595, 525, 493. The peaks 74 and 75 were tentatively characterized as (*E*)-aldosecologanin, (*Z*)-aldosecologanin. The peaks (89, 90 and 91) both presented [M+H]^+^ at 405.1393. In the MS/MS spectrum, they produced fragment ions at *m/z* 243, 211, 193. The peaks 89, 90 and 91 were tentatively characterized as secoxyloganin and its isomers. The peaks (92 and 93) both presented [M+H]^+^ at 391.1600. In the MS/MS spectrum, they produced fragment ions at *m/z* 229, 211, 197. The peaks 92 and 93 were tentatively characterized as loganin and its isomer. Among the iridoids, 43 were detected in the flowers and flower buds, 41 in the stems and 32 in the leaves. Lonijapospiroside B I, lonijapospiroside B II, lonijapospiroside B III, secoxyloganin I, secoxyloganin II, loganin II, 5α-carboxystrictosidine I, 5α-carboxystrictosidine III, 7-*O*-ethylsweroside, lonijaposide T, dimethyl lonijaposide C were missing in the leaves. Missing in the stems were 5α-carboxystrictosidine I, 5α-carboxystrictosidine II.

### 3.2. Metabolomics Difference in Different Tissues of LJT

**Figure 2:**
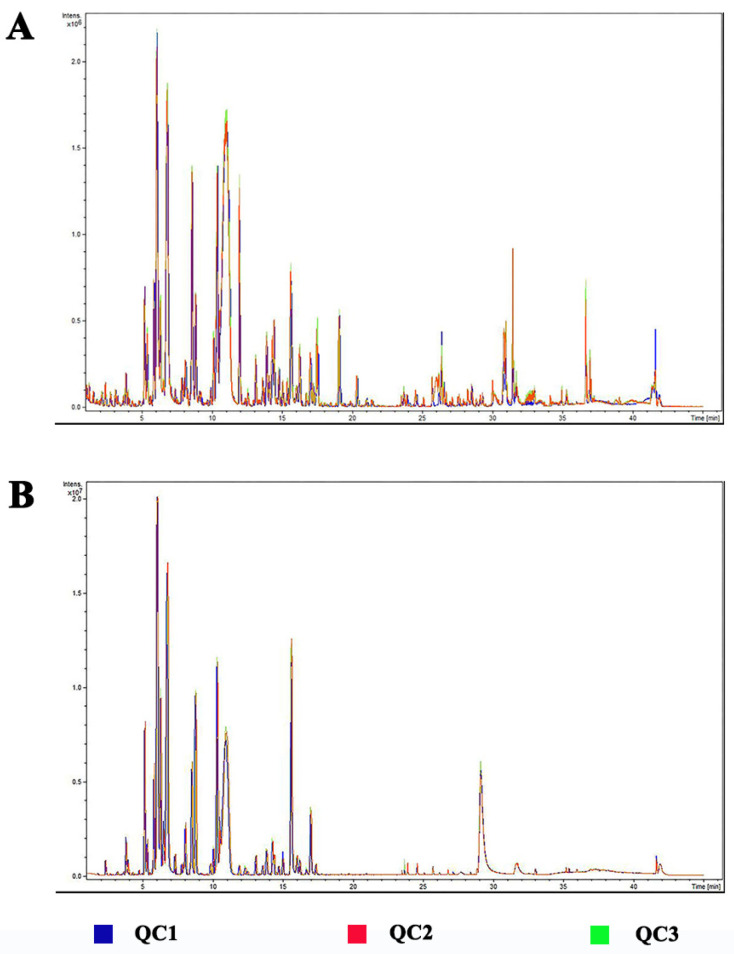
The total ion current chromatograms (TICs) of the QC samples. (**A**) positive ion mode (ESI+). (**B**) negative ion mode (ESI−).

A total of 3 QC samples were prepared and analyzed. The total ion current chromatograms (TICs) of the QC samples in positive ion mode (ESI+) and negative ion mode (ESI-) are shown in Figure 2 respectively. It was found that peak intensity and retention time basically overlapped, which means that the perturbation during the process of the experiment is small. The relative standard deviation (RSD) of each peak area was calculated. The peak number and accumulated peak area were counted within different RSD ranges (0−10, 10−20, 20−30, and >30%). The 98.3% of the peaks have RSD less than 20%, accounting for 99.8% of the total area. It indicates good repeatability and stability of the metabolomic analysis.

**Figure 3:**
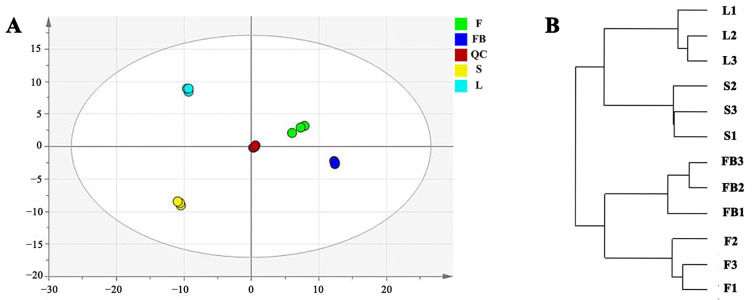
Analysis of metabolic variation in different tissues of LJT. (**A**) the score plot of PCA anal-ysis. (**B**) hierarchical cluster analysis of different tissues of LJT.

Principal component analysis was performed to find the metabolomic differences in different tissues of LJT. It was found that two principal components explained 71.90% of the overall variance (51.00% and 20.90% for PC1 and PC2 respectively, Figure 3A). There were significant variations among the four tissues, since they dispersed in four different parts of the score plot. Hierarchical cluster analysis was then carried out for the four tissues. Samples of flower buds, flowers, leaves and stems of LJT formed four separate clusters (Figure 3B). Moreover, the samples of leaves and stems clustered together, and the samples of flower buds and flowers clustered together, suggesting a similarity in the composition and content.

Orthogonal partial least squares-discrimination analysis was performed between each two groups of samples. The score plot showed significant differences among them. Differential metabolites were filtered by VIP value (VIP > 1) and fold-change (FC ≥ 4). There were 27 significantly differential metabolites between flowers and flower buds (18 up-regulated, 9 down-regulated), 81 between leaves and flower buds (69 up-regulated, 12 down-regulated), 113 between stems and flower buds (106 up-regulated, 7 down-regulated), 66 between leaves and flowers (54 up-regulated, 12 down-regulated), 98 between stems and flowers (93 up-regulated, 5 down-regulated), 79 between stems and leaves (58 up-regulated, 21 down-regulated). Most of these differential metabolites were secondary metabolites.

Kyoto Encyclopedia of Genes and Genomes is one of the databases for pathway research, which contains pathway information on metabolism, genetic information processing [24]. In the current study, differential metabolites were annotated by the KEGG database. Through the KEGG pathway analysis, it was found that the differential metabolites were mainly enriched in five pathways, including flavone and flavonol biosynthesis (Ko00944), flavonoid biosynthesis (Ko00941), phenylpropanoid biosynthesis (Ko00940), biosynthesis of amino acids (Ko01230), monoterpenoid biosynthesis (Ko00902), citrate cycle (Ko00020). There were more differential metabolites enriched in the phenylpropanoid biosynthesis pathway than those in other pathways. The phenylpropanoid biosynthesis pathway is an indispensable pathway which provides precursors for many secondary metabolites. Phenylpropanoid biosynthesis mainly includes the biosynthesis of amino acids, flavonoids and phenolic acids. Subsequently, heat map analysis was carried out for the 132 differential metabolites, and metabolites dynamic changes were analyzed using the biosynthesis pathway map.

#### 3.2.1. Differences in Primary Metabolites of Different Tissues

**Figure 4:**
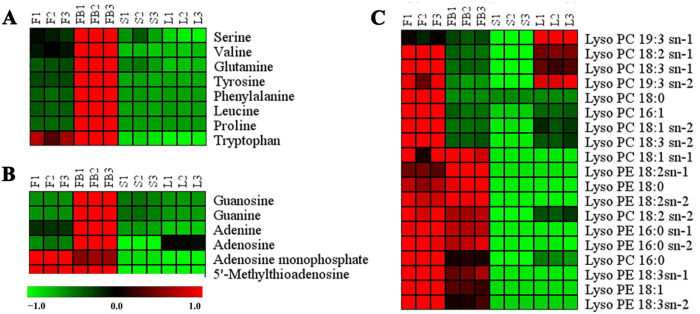
Heat map of differential primary metabolites. (**A**) amino acids. (**B**) nucleosides. (**C**) lipids. Red represents higher content and green represents lower content.

Figure 4 is the heat map of differential primary metabolites. Amino acids were significantly more abundant in the flower buds than other tissues. Amino acids are critical to plant development. They play the role as precursors or nitrogen donors for the synthesis of nucleotides, hormones and secondary metabolites [25]. Phenylalanine and tryptophan are important precursors for the synthesis of phenolic acids and flavonoids in the phenylpropanoid biosynthesis pathway [26]. The amount of phenylalanine and tryptophan in flowers buds was higher than in other tissues, which was related to the distributions of flavonoids and phenolic acids. The amount of phenylalanine in flower buds was 7.52-fold, 31.91-fold and 33.83-fold more than in the flowers, stems and leaves, respectively. Similarly, the amount of tryptophan in flower buds was 5.23-fold, 32.25-fold and 35.46-fold more than in the flowers, stems and leaves, respectively.

Nucleotides are important metabolites of plant growth and development [27]. The amount of nucleotides in the flower buds was much higher than in other tissues (Figure 4B). Lipids are essential constituents of plant cells. They provide structural integrity and energy for various metabolic processes [28]. As is shown in Figure 4C, the amount of lipids was significantly higher in the flowers than that in the other tissues.

#### 3.2.2. Differences in Secondary Metabolites of Different Tissues

Phenylalanine and tryptophan are catalyzed by enzymes to form cinnamic acid and p-coumaroyl-CoA. Then the phenylpropanoid pathway divides into two branches: the phenylalanine metabolic pathway and the flavonoid metabolic pathway [29]. Flavonoid biosynthesis is an important downstream branch of phenylpropanoid metabolism.

**Figure 5:**
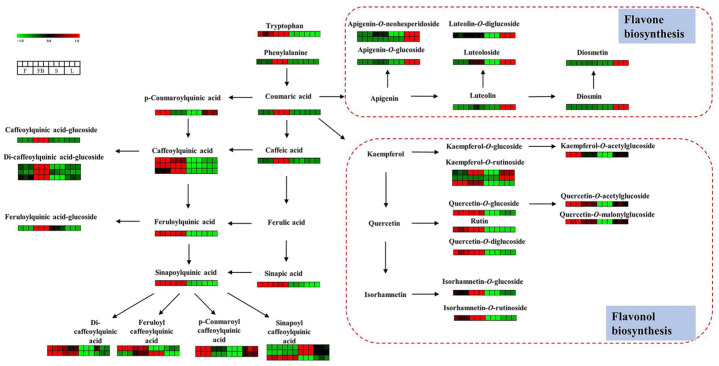
Visualization of phenolic acids and flavonoids in biosynthesis pathway map. Red box represents a higher content, and green box represents a lower content.

As is shown in Figure 5, there are significant differences in flavonoids in different tissues of LJT. Most flavones showed higher expression in leaves, such as apigenin-*O*-neohesperidoside, apigenin-7-*O*-glucoside, luteolin, luteoloside, luteolin-*O*-diglucoside, diosmin and diosmetin-*O*-glucoside. Flavonoids that accumulated in leaves mainly involved the flavone biosynthesis pathway. The content of luteolin in the leaves was 5.27-fold, 12.23-fold and 12.47-fold higher than that in flower buds, flowers and stems, respectively. The content of luteoloside in the leaves was 1.74-fold, 3.51-fold and 135.81-fold higher than that in flower buds, flowers and stems, respectively. Similarly, the content of apigenin-7-*O*-glucoside in the leaves was 6.39-fold and 11.82-fold higher than that in the flower buds and flowers. The content of apigenin-*O*-neohesperidoside in the leaves was 82.86-fold, 69.01-fold and 456.12-fold higher than that in the flower buds, flowers and stems. Moreover, the content of diosmin in the leaves was 23.05-fold and 22.33-fold higher than that in flowers buds and flowers. The content of diosmetin in leaves was 20.81-fold and 36.34-fold higher than that in flowers buds and flowers. Flavones are bioavailable molecules with antioxidant, anti-inflammatory, antibacterial, antiviral and anticancer activities [19]. Luteolin and luteoloside, as the most common flavones, have been reported to inhibit the transcriptional activation of STAT3/IRF-1, NF-κB and AP-1 pathways and exert anti-inflammatory effects [30]. Apigenin-7-*O*-glucoside can protect free radical-induced oxidative damage of pBR322 DNA, bovine serum albumin and erythrocytes [31]. Most flavonols showed higher expression in the flower buds, including quercetin-3-*O*-glucoside, quercetin-*O*-diglucoside, rutin, isorhamnetin-3-*O*-glucoside, isorhamnetin-*O*-rutinoside, kaempferol-*O*-rutinoside. Flavonoids which accumulated in the flower buds mainly involved the flavonol biosynthesis pathway. The amount of isorhamnetin-3-*O*-glucoside in the flower buds was 2.06-fold and 4.32-fold higher than that in the flowers and leaves. The amount of quercetin-3-*O*-glucoside in the flower buds was 1.04-fold, 3.17-fold and 75.60-fold higher than that in the flowers, leaves and stems, respectively. The amount of rutin in the flower buds was 1.31-fold, 37.75-fold and 635.04-fold higher than that in the flowers, leaves and stems, respectively. The kaempferol-*O*-rutinoside in the flower buds was 2.23-fold, 1.21-fold, 129.02-fold higher than that in the flowers, leaves and stems. Flavonols also have beneficial activities. Isorhamnetin-3-*O*-glucoside can effectively protect lens protein by maintaining Ca^2+^-ATPase activity, preventing oxidative stress, calcium accumulation and lipid peroxidation [32]. Quercetin-3-*O*-glucoside inhibits pancreatic cancer cell migration and has an anti-cancer effect [33]. Moreover, flavonols can filter some harmful solar wave-lengths to protect DNA [34]. For acylated flavonoid glucosides, kaempferol-*O*-acetylglucoside, quercetin-*O*-acetylglucoside, quercetin-*O*-malonylglucoside were abundant in the flowers. The amount of kaempferol-acetylglucoside in flowers was 2.11-fold, 1.81-fold and 20.01-fold higher than that in flower buds, leaves and stems, respectively. The amount of quercetin-acetylglucoside in flowers was 1.27-fold, 1.45-fold and 16.21-fold higher than that in the flower buds, leaves and stems, respectively. The amount of quercetin-malonylglucoside in the flowers was 1.30-fold, 1.47-fold and 15.60-fold higher than that in the flower buds, leaves and stems, respectively. Acylation of flavonoids can further affect their physicochemical properties and pharmacological effects. Acylation reaction of flavonoids with aromatic carboxylic acids markedly enhances antioxidant activities [35,36].

Twenty-six phenolic acids with significant differential expression were found among different tissues. As shown in Figure 5, most phenolic acids showed a higher expression in the flower buds and flowers. Free phenolic acids, including coumaric acid, caffeic acid, ferulic acid, were much higher in the flower buds than those in other tissues. The amount of coumaric acid in the flower buds was 9.75-fold, 19.56-fold and 46.03-fold higher than that in the flowers, leaves and stems, respectively. The amount of caffeic acid in the flower buds was 4.18-fold, 5.36-fold, 1.58-fold higher than that in the flowers, leaves and stems, respectively. Caffeic acid can kill a diverse range of microbial pathogens, exerting an antimicrobial effect [37]. Bound phenolic acids were conjugated with quinic acid or glucose. Most bound phenolic acids were more abundant in the flowers and flower buds, including caffeoylquinic acid, di-caffeoylquinic acid, feruloylquinic acid, sinapoylquinic acid, feruloyl caffeoylquinic acid, etc. For example, the amount of caffeoylquinic acid isomers in the flower buds was 0.77-2.45 times, 2.17-30.01 times and 3.32-212.34 times more than in the flowers, leaves and stems, respectively. The amount of di-caffeoylquinic acid isomers in the flower buds was 0.61-1.06 times, 1.49-2.43 times and 3.31-25.93 times more than in the flowers, leaves and stems, respectively. The amount of feruloylquinic acid in the flower buds was 1.05-fold, 21.75-fold and 5.61-fold higher than in the flowers, leaves and stems, respectively. The amount of sinapoylquinic acid in the flower buds was 1.05-fold, 21.75-fold and 5.61-fold higher than that in flowers, leaves and stems, respectively. Caffeoylquinic acid and its derivatives are the main active ingredients of LJT, which have many therapeutic effects, such as antioxidant activity, antibacterial, liver protection, heart protection, anti-inflammatory, neuroprotective, anti-obesity, antiviral, anti-microbial, anti-hypertension [38]. The amount of phenolic acid-glucosides was higher in the flower buds than in other tissues such as caffeoylquinic acid-glucoside, feruloylquinic acid-glucoside, di-caffeoylquinic acid-glucoside. The amount of caffeoylquinic acid-glucoside in the flower buds was 7.10-fold, 6.20-fold, 6.65-fold higher than that in the flowers, leaves and stems, respectively. Similarly, the amount of feruloylquinic acid-glucoside in the flower buds was 7.07-fold, 7.68-fold, 2.66-fold higher than in the flowers, leaves and stems respectively. The amount of di-caffeoylquinic acid-glucoside isomers in the flower buds was 2.13–5.82 times, 2.74–7.56 and 4.74–30.88 times more than in the flowers, leaves and stems, respectively. Di-caffeoylquinic acid glucoside has a significant protective effect on DNA damage induced by ROO and OH radicals [39].

**Figure 6:**
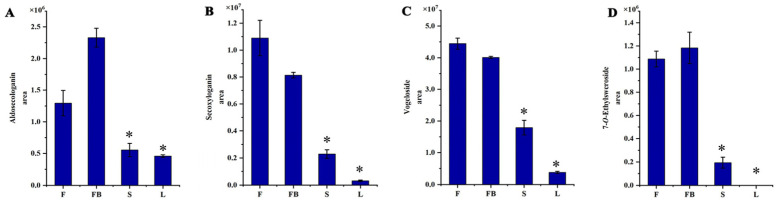
The content of iridoids in different tissues of LJT. (**A**) Aldosecologanin. (**B**) Secologanin, (**C**) Vogeloside. (**D**) 7-*O*-ethylsweroside. * means that significant difference was found between FB/S and FB/L.

Thirty-two iridoids with significant differential expression were found among different tissues of LJT. As shown in Figure S1, most iridoids showed a higher expression in the flowers and flower buds. Aldosecologain, secoxyloganin, vogeloside, 7-*O*-ethylsweroside were the most abundant iridoids in the LJT (Figure 6). The amount of secoxyloganin in the flowers was 1.34-fold, 34.4-fold and 4.77-fold higher than that in the flower buds, leaves and stems, respectively. The amount of vogeloside in the flowers was 1.10-fold, 11.81-fold and 2.46-fold higher than that in the flower buds, leaves and stems, respectively. The amount of aldosecologanin in the flower buds was 1.80-fold, 5.08-fold and 4.20-fold higher than that in the flowers, leaves and stems, respectively. The amount of 7-*O*-ethylsweroside in the flower buds was 1.09-fold and 6.10-fold higher than that in the flowers and stems, respectively. However, 7-*O*-ethylsweroside was not detected in leaves. Iridoids have many pharmacological activities such as liver protection, anti-inflammatory and anti-cancer effects [40]. Vogeloside can inhibit nitric oxide production by lipopolysaccharide-activated macrophages, play a significant anti-inflammatory effect [41]. The amount and composition of flavonoids, phenolic acids and iridoids was significantly different in the four medicinal tissues, so the pharmacological effects and action mechanisms may also be different among them.

## 4. Conclusions

In this study, flowers, flower buds, stems and leaves of LJT were analyzed with un-targeted metabolomics based on UHPLC-Q-TOF MS. The major active components were identified in different tissues of LJT, including flavonoids, phenolic acids and iridoids. The content of metabolites in four tissues were significantly different. Flower buds were rich in flavonols, phenolic acids and iridoids. Flowers contained more acylated flavonol glucosides, phenolic acids and iridoids. Leaves were rich in flavones, and most metabolites showed lower content in the stems. The changes in metabolite content and composition can lead to differences in pharmacological effects and mechanisms among the four different tissues. The detailed relationship between the differential metabolites and their pharmacological activities needs further investigation.

## Figures and Tables

**Table 1:** Identification of secondary metabolites in different tissues of LJT.

No.	Name	t_R_/min	*m/z*	Formula	Classification	Mode	MS Fragments	Tissues
1	Apigenin-5-*O*-neohesperidoside	15.59	577.1558	C_27_H_30_O_14_	Flavonoids	[M−H]^−^	431, 269, 251, 223	F/FB/L
2	Apigenin-7-*O*-neohesperidoside	16.30	577.1558	C_27_H_30_O_14_	Flavonoids	[M−H]^−^	431, 269, 251, 223	F/FB/S/L
3	Apigenin-7-glucoside	16.23	431.0976	C_21_H_20_O_10_	Flavonoids	[M−H]^−^	269, 251, 223	F/FB/L
4	Isorhamnetin-3-*O*-glucoside	16.25	477.1030	C_22_H_22_O_12_	Flavonoids	[M−H]^−^	315, 300, 271, 243, 179, 151	F/FB/L
5	Luteoloside	13.93	447.0924	C_21_H_20_O_11_	Flavonoids	[M−H]^−^	285, 241, 151, 133	F/FB/S/L
6	Quercetin-3-*O*-glucoside	13.65	463.0874	C_21_H_20_O_12_	Flavonoids	[M−H]^−^	301, 300, 271, 255, 243, 151	F/FB/S/L
7	Isorhamnetin-*O*-rutinoside Ⅰ	15.66	623.1608	C_28_H_32_O_16_	Flavonoids	[M−H]^−^	315, 299, 271, 243, 178, 151	F/FB/L
8	Isorhamnetin-*O*-rutinoside Ⅱ	16.30	623.1608	C_28_H_32_O_16_	Flavonoids	[M−H]^−^	315, 299, 271, 243, 178, 151	F/FB/L
9	Luteolin-*O*-diglucoside	10.42	609.1452	C_27_H_30_O_16_	Flavonoids	[M−H]^−^	285, 199, 151, 133	F/FB/L
10	Kaempferol-*O*-rutinoside Ⅰ	13.58	593.1502	C_27_H_30_O_15_	Flavonoids	[M−H]^−^	285, 255	F/FB/S/L
11	Kaempferol-*O*-rutinoside Ⅱ	14.35	593.1502	C_27_H_30_O_15_	Flavonoids	[M−H]^−^	285, 255	F/FB/S/L
12	kaempferol-*O*-rutinoside Ⅲ	15.12	593.1502	C_27_H_30_O_15_	Flavonoids	[M−H]^−^	285, 255	F/FB/L
13	Quercetin-*O*-diglucoside	7.51	625.1396	C_27_H_30_O_17_	Flavonoids	[M−H]^−^	301, 271, 213, 193, 150, 117	F/FB/S/L
14	Quercetin-7-*O*-rutinosie	8.10	609.1447	C_27_H_30_O_16_	Flavonoids	[M−H]^−^	301, 271, 255, 178	F/FB/S/L
15	Rutin	13.16	609.1447	C_27_H_30_O_16_	Flavonoids	[M−H]^−^	300, 271, 255, 178, 151	F/FB/S/L
16	Diosmin	17.02	607.1658	C_28_H_32_O_15_	Flavonoids	[M−H]^−^	300, 299, 284	F/FB/L
17	Luteolin	21.30	285.0395	C_15_H_10_O_6_	Flavonoids	[M−H]^−^	151, 133, 121, 107	F/FB/S/L
18	Kaempferol-*O*-acetylglucoside	17.30	489.1030	C_23_H_22_O_12_	Flavonoids	[M−H]^−^	285, 255, 227, 153	F/FB/S/L
19	Quercetin-*O*-acetylglucoside	14.89	505.0975	C_23_H_22_O_13_	Flavonoids	[M−H]^−^	300, 271, 255, 234, 179, 151	F/FB/S/L
20	Quercetin-*O*-malonylglucoside	14.89	549.0873	C_24_H_22_O_15_	Flavonoids	[M−H]^−^	300, 271, 255, 234, 179, 151	F/FB/S/L
21	Diosmetin	24.12	299.0548	C_16_H_12_O_6_	Flavonoids	[M−H]^−^	269, 151	F/FB/L
22	Apigenin	29.81	269.0447	C_15_H_10_O_5_	Flavonoids	[M−H]^−^	251, 241, 223	F/FB/S/L
23	Diosmetin-*O*-glucoside Ⅰ	17.10	463.1236	C_22_H_22_O_11_	Flavonoids	[M+H]^+^	301, 286, 258, 153	F/FB/S/L
24	Diosmetin-*O*-glucoside Ⅱ	17.70	463.1236	C_22_H_22_O_11_	Flavonoids	[M+H]^+^	301, 286, 258, 153	F/FB/S/L
25	5-Hydroxyl-3’,4’,7-trimethoxy flavone	28.60	329.1018	C_18_H_16_O_6_	Flavonoids	[M+H]^+^	314, 313	F/FB/S
26	Corymbosin	29.00	359.1123	C_19_H_18_O_7_	Flavonoids	[M+H]^+^	329	F/FB
27	Flavoyadorinin B	23.30	477.1390	C_23_H_24_O_11_	Flavonoids	[M+H]^+^	315, 300	F/FB
28	Eriodictryol-7-*O*-glucoside	13.93	449.1080	C_21_H_22_O_11_	Flavonoids	[M−H]^−^	415, 315, 299, 298, 163	F/FB/S/L
29	Neochlorogenic acid	3.94	353.0869	C_16_H_18_O_9_	Phenolic acids	[M−H]^−^	191, 179, 163, 145, 135	F/FB/S/L
30	Chlorogenic acid	6.16	353.0869	C_16_H_18_O_9_	Phenolic acids	[M−H]^−^	191, 179, 163, 145, 135	F/FB/S/L
31	Cryptochlorogenic acid	8.15	353.0869	C_16_H_18_O_9_	Phenolic acids	[M−H]^−^	191, 179, 173, 163, 145	F/FB/S/L
32	3-*O*-p-Coumaroylquinic acid	8.52	337.0915	C_16_H_18_O_8_	Phenolic acids	[M−H]^−^	191, 173, 163, 93	F/FB/S/L
33	4-*O*-p-Coumaroylquinic acid	10.44	337.0915	C_16_H_18_O_8_	Phenolic acids	[M−H]^−^	191, 173, 163, 93	F/FB/S/L
34	3-*O*-Feruloylquinic acid	10.81	367.1025	C_17_H_20_O_9_	Phenolic acids	[M−H]^−^	193, 191, 173, 93	F/FB/S/L
35	4-*O*-Feruloylquinic acid	11.54	367.1025	C_17_H_20_O_9_	Phenolic acids	[M−H]^−^	193, 191, 173, 93	F/FB/S/L
36	Sinapoylquinic acid	10.42	397.1130	C_18_H_22_O_10_	Phenolic acids	[M−H]^−^	223, 191, 173	F/FB/S/L
37	Isochlorogenic acid B	15.10	515.1186	C_25_H_24_O_12_	Phenolic acids	[M−H]^−^	353, 191, 179, 173	F/FB/S/L
38	Isochlorogenic acid A	15.68	515.1186	C_25_H_24_O_12_	Phenolic acids	[M−H]^−^	353, 191, 179, 173	F/FB/S/L
39	Isochlorogenic acid C	17.06	515.1186	C_25_H_24_O_12_	Phenolic acids	[M−H]^−^	353, 191, 179, 173	F/FB/S/L
40	Feruloylcaffeoylquinic acid Ⅰ	19.23	529.1338	C_26_H_26_O_12_	Phenolic acids	[M−H]^−^	367, 353, 173	F/FB/S/L
41	Feruloylcaffeoylquinic acid Ⅱ	20.01	529.1338	C_26_H_26_O_12_	Phenolic acids	[M−H]^−^	367, 353, 173	F/FB/S/L
42	Feruloylcaffeoylquinic acid Ⅲ	20.38	529.1338	C_26_H_26_O_12_	Phenolic acids	[M−H]^−^	367, 353, 173	F/FB/S/L
43	p-Coumaroyl caffeoylquinic acid Ⅰ	18.22	499.1230	C_25_H_24_O_11_	Phenolic acids	[M−H]^−^	353, 337, 335	F/FB/S/L
44	p-Coumaroyl caffeoylquinic acid Ⅱ	18.65	499.1230	C_25_H_24_O_11_	Phenolic acids	[M−H]^−^	353, 337, 335	F/FB/S/L
45	p-Coumaroyl caffeoylquinic acid Ⅲ	19.77	499.1230	C_25_H_24_O_11_	Phenolic acids	[M−H]^−^	353, 337, 335	F/FB/S/L
46	Sinapoyl caffeoylquinic acid Ⅰ	16.88	559.1448	C_27_H_28_O_13_	Phenolic acids	[M−H]^−^	397, 353, 223, 173	F/FB/S/L
47	Sinapoyl caffeoylquinic acid Ⅱ	17.44	559.1448	C_27_H_28_O_13_	Phenolic acids	[M−H]^−^	397, 353, 223, 173	F/FB/S/L
48	Sinapoyl caffeoylquinic acid Ⅲ	19.68	559.1448	C_27_H_28_O_13_	Phenolic acids	[M−H]^−^	397, 353, 223, 173	F/FB/S/L
49	Caffeoylquinic acid-glucoside	3.78	515.1397	C_22_H_28_O_14_	Phenolic acids	[M−H]^−^	353, 191, 179	F/FB/S/L
50	Feruloylquinic acid-glucoside	4.98	529.1555	C_23_H_30_O_14_	Phenolic acids	[M−H]^−^	367, 193, 191	F/FB/S/L
51	Sinapoylquinic acid-glucoside	10.69	559.1659	C_24_H_32_O_15_	Phenolic acids	[M−H]^−^	397, 223, 191	F/FB/S/L
52	3,4-*O*-caffeoylquinic acid-glucoside	11.64	677.1920	C_28_H_38_O_19_	Phenolic acids	[M−H]^−^	515, 353, 191	F/FB/S/L
53	3,5-*O*-caffeoylquinic acid-glucoside	12.74	677.1920	C_28_H_38_O_19_	Phenolic acids	[M−H]^−^	515, 353, 191	F/FB/S/L
54	4,5-*O*-caffeoylquinic acid-glucoside	13.48	677.1920	C_28_H_38_O_19_	Phenolic acids	[M−H]^−^	515, 353, 191	F/FB/S/L
55	p-Coumaroyl-glucoside	6.23	325.0919	C_15_H_18_O_8_	Phenolic acids	[M−H]^−^	163	F/FB/S/L
56	Protocatechuic acid-4-glucoside	2.54	315.0712	C_13_H_16_O_9_	Phenolic acids	[M−H]^−^	153	F/FB/S/L
57	Isoeugenol	20.52	163.0756	C_10_H_12_O_2_	Phenolic acids	[M−H]^−^	148	F/FB/S/L
58	Cinnamic acid	28.9	149.0598	C_9_H_8_O_2_	Phenolic acids	[M+H]^+^	131, 103	F/FB/S/L
59	Caffeic acid	5.80	181.0496	C_9_H_8_O_4_	Phenolic acids	[M+H]^+^	163, 145, 89	F/FB/S/L
60	Sinapic acid	10.50	225.0759	C_11_H_12_O_5_	Phenolic acids	[M+H]^+^	207, 175	F/FB/S/L
61	Ferulic acid	6.90	195.0654	C_10_H_10_O_4_	Phenolic acids	[M+H]^+^	177, 145	F/FB/S/L
62	Coumaric acid	6.20	165.0548	C_9_H_8_O_3_	Phenolic acids	[M+H]^+^	147, 119	F/FB/S/L
63	3-Hydroxybenzoic acid	5.14	137.0234	C_7_H_6_O_3_	Phenolic acids	[M−H]^−^	137, 93	F/FB/S/L
64	Lonijapospiroside B Ⅰ	16.72	560.1764	C_27_H_31_NO_12_	Iridoids	[M−H]^−^	398, 380, 328, 296, 284	F/FB/S
65	Lonijapospiroside B Ⅱ	19.42	560.1764	C_27_H_31_NO_12_	Iridoids	[M−H]^−^	398, 380, 328, 296, 284	F/FB/S
66	Lonijapospiroside B Ⅲ	20.92	560.1764	C_27_H_31_NO_12_	Iridoids	[M−H]^−^	398, 380, 328, 296, 284	F/FB/S
67	Morroniside Ⅰ	8.62	405.1393	C_17_H_26_O_11_	Iridoids	[M−H]^−^	373, 225, 179	F/FB/S/L
68	Morroniside Ⅱ	10.42	405.1393	C_17_H_26_O_11_	Iridoids	[M−H]^−^	373, 225, 179	F/FB/S/L
69	Secologanoside Ⅰ	2.48	389.1081	C_16_H_22_O_11_	Iridoids	[M−H]^−^	209, 165, 121	F/FB/S/L
70	Secologanoside Ⅱ	3.33	389.1081	C_16_H_22_O_11_	Iridoids	[M−H]^−^	209, 165, 121	F/FB/S/L
71	Secologanoside Ⅲ	6.40	389.1081	C_16_H_22_O_11_	Iridoids	[M−H]^−^	209, 165, 121	F/FB/S/L
72	Dimethylsecologanoside Ⅰ	11.57	417.1394	C_18_H_26_O_11_	Iridoids	[M−H]^−^	255, 237, 185, 163, 155	F/FB/S/L
73	Dimethylsecologanoside Ⅱ	13.52	417.1394	C_18_H_26_O_11_	Iridoids	[M−H]^−^	255, 237, 185, 163, 155	F/FB/S/L
74	(*E*)-Aldosecologanin	16.77	757.2550	C_34_H_46_O_19_	Iridoids	[M−H]^−^	595, 525, 493	F/FB/S/L
75	(*Z*)-Aldosecologanin	17.83	757.2550	C_34_H_46_O_19_	Iridoids	[M−H]^−^	595, 525, 493	F/FB/S/L
76	Swertiamarin	6.86	373.1131	C_16_H_22_O_10_	Iridoids	[M−H]^−^	211, 193, 167, 149, 123	F/FB/S/L
77	Genameside A Ⅰ	3.58	421.1342	C_17_H_26_O_12_	Iridoids	[M−H]^−^	241, 197	F/FB/S/L
78	Genameside A Ⅱ	4.09	421.1342	C_17_H_26_O_12_	Iridoids	[M−H]^−^	241, 197	F/FB/S/L
79	Genameside A Ⅲ	5.27	421.1342	C_17_H_26_O_12_	Iridoids	[M−H]^−^	241, 197	F/FB/S/L
80	8-Epi-loganic acid	4.41	375.1288	C_16_H_24_O_10_	Iridoids	[M−H]^−^	213, 169, 151, 125	F/FB/S/L
81	7-Epi-loganic acid	5.27	375.1288	C_16_H_24_O_10_	Iridoids	[M−H]^−^	213, 169, 151, 125	F/FB/S/L
82	Arbutoside Ⅰ	7.60	697.2189	C_28_H_42_O_20_	Iridoids	[M−H]^−^	535, 373, 355, 341	F/FB/S/L
83	Arbutoside Ⅱ	8.04	697.2189	C_28_H_42_O_20_	Iridoids	[M−H]^−^	535, 373, 355, 341	F/FB/S/L
84	Demethyl-strychoside A	12.41	729.2239	C_32_H_42_O_19_	Iridoids	[M−H]^−^	549, 505, 497, 453, 409	F/FB/S/L
85	7-*O*-Methyl morroniside	9.86	419.1549	C_18_H_28_O_11_	Iridoids	[M−H]^−^	239	F/FB/S/L
86	Strychoside A Ⅰ	14.15	743.2396	C_33_H_44_O_19_	Iridoids	[M−H]^−^	581, 563, 511, 467	F/FB/S/L
87	Strychoside A Ⅱ	14.65	743.2396	C_33_H_44_O_19_	Iridoids	[M−H]^−^	581, 563, 511, 467	F/FB/S/L
88	Loganic acid-*O*-pentoside	7.50	509.1868	C_21_H_32_O_14_	Iridoids	[M+H]^+^	377	F/FB/S/L
89	Secoxyloganin Ⅰ	5.50	405.1393	C_17_H_24_O_11_	Iridoids	[M+H]^+^	243, 211, 193, 167	F/FB/S
90	Secoxyloganin Ⅱ	7.10	405.1393	C_17_H_24_O_11_	Iridoids	[M+H]^+^	243, 211, 193, 167	F/FB/S
91	Secoxyloganin Ⅲ	10.50	405.1393	C_17_H_24_O_11_	Iridoids	[M+H]^+^	243, 211, 193, 167	F/FB/S/L
92	Loganin Ⅰ	8.90	391.1600	C_17_H_26_O_10_	Iridoids	[M+H]^+^	229. 211, 197, 193, 179, 167	F/FB/S/L
93	Loganin Ⅱ	10.20	391.1600	C_17_H_26_O_10_	Iridoids	[M+H]^+^	229. 211, 197, 193, 179, 167	F/FB/S
94	5α-Carboxystrictosidine Ⅰ	12.80	575.2232	C_28_H_34_N_2_O_11_	Iridoids	[M+H]^+^	413, 395, 343	F/FB
95	5α-Carboxystrictosidine Ⅱ	14.40	575.2232	C_28_H_34_N_2_O_11_	Iridoids	[M+H]^+^	413, 395, 343	F/FB/L
96	5α-Carboxystrictosidine Ⅲ	15.60	575.2233	C_28_H_34_N_2_O_11_	Iridoids	[M+H]^+^	413, 395, 343	F/FB/S
97	5α-Carboxystrictosidine Ⅳ	17.40	575.2232	C_28_H_34_N_2_O_11_	Iridoids	[M+H]^+^	413, 395, 343	F/FB/S/L
98	Hydro-dimethyl lonijaposide C	8.40	554.2234	C_26_H_35_NO_12_	Iridoids	[M+H]^+^	392, 374	F/FB/S/L
99	Vogeloside Ⅰ	11.20	389.1445	C_17_H_24_O_10_	Iridoids	[M+H]^+^	227, 209, 195, 177, 151	F/FB/S/L
100	Vogeloside Ⅱ	10.40	389.1445	C_17_H_24_O_10_	Iridoids	[M+H]^+^	227, 209, 195, 177, 151	F/FB/S/L
101	7-*O*-Ethylsweroside	15.20	403.1596	C_18_H_26_O_10_	Iridoids	[M+H]^+^	241, 209, 177	F/FB/S
102	Secologanic acid	6.90	375.1287	C_16_H_22_O_10_	Iridoids	[M+H]^+^	213, 195, 151	F/FB/S/L
103	Lonijaposide T	10.50	594.2184	C_28_H_35_NO_13_	Iridoids	[M+H]^+^	362	F/FB/S
104	Lonijaposide B	12.10	538.1920	C_25_H_31_NO_12_	Iridoids	[M+H]^+^	376, 358, 344, 211	F/FB/S/L
105	Dimethyl lonijaposide C	8.90	552.2078	C_26_H_33_NO_12_	Iridoids	[M+H]^+^	390, 320,288	F/FB/S
106	Sweroside	8.70	359.1339	C_16_H_22_O_9_	Iridoids	[M+H]^+^	197, 179, 127	F/FB/S/L

## Data Availability

The data are available in the article and supplementary material.

## Supplementary Materials

The following supporting information can be downloaded at: https://www.mdpi.com/article/10.3390/metabo13020186/s1, Table S1: Identification of primary metabolites in LJT. Figure S1: Heat map of differential iridoids in four different tissues of LJT.
