# Supplementary material for: Metabolomics Analysis of Different Tissues of Lonicera japonica Thunb. Based on Liquid Chromatography with Mass Spectrometry
Source: Metabolites. 2023 Jan 27;13(2):186. doi: 10.3390/metabo13020186 (PMC9964630; doi:10.3390/metabo13020186)
Supplement: Supplementary file 1 [file metabolites-13-00186-s001.zip › metabolites-2185133-supplementary.pdf]

Supplementary Materials

Metabolomics analysis of different tissues of *Lonicera japonica* Thunb. based on liquid chromatography with mass spectrometry

Yan Wang [1, 2], Lili Li [1, 3*], Wenhua Ji [1, 3], Shuang Liu [1, 3], Jiali Fan [1, 3], Heng Lu [1, 3], Xiao Wang [1, 3*]

[1] Key Laboratory for Applied Technology of Sophisticated Analytical Instruments of Shandong Province, Shandong Analysis and Test Center, Qilu University of Technology, Shandong Academy of Sciences, Jinan 250014, China
[2] College of Traditional Chinese Medicine, Yunnan University of Chinese Medicine, Kunming 650000, China
3 School of Pharmaceutical Sciences, Qilu University of Technology, Shandong Academy of Sciences, Jinan 250014, China
* Correspondence: correspondence to: Lili Li, liliouc@126.com; Xiao Wang, wangx@sdas.org.

Table S1. Identification of primary metabolites in LJT.

No.	Name	t_R/min	*m/z*	Classification	Mode
1	Glutamine	0.70	147.0760	Amino acids	$[M+H]^+$
2	Serine	0.70	106.0500	Amino acids	$[M+H]^+$
3	Phenylalanine	2.10	166.0860	Amino acids	$[M+H]^+$
4	Tyrosine	0.90	182.0810	Amino acids	$[M+H]^+$
5	Valine	0.90	118.0860	Amino acids	$[M+H]^+$
6	Leucine	1.20	132.1020	Amino acids	$[M+H]^+$
7	Glutamic acid	0.70	148.0600	Amino acids	$[M+H]^+$
8	Proline	0.80	116.0700	Amino acids	$[M+H]^+$
9	Tryptophan	4.00	205.0970	Amino acids	$[M+H]^+$
10	Adenosine monophosphate	1.00	348.0710	Nucleotides	$[M+H]^+$
11	Guanosine	1.30	284.0990	Nucleotides	$[M+H]^+$
12	Guanine	1.30	152.0570	Nucleotides	$[M+H]^+$
13	Adenine	1.20	136.0620	Nucleotides	$[M+H]^+$
14	Adenosine	1.20	268.1040	Nucleotides	$[M+H]^+$
15	5'-Methylthioadenosine	4.30	298.0970	Nucleotides	$[M+H]^+$
16	FA 16:1	34.11	253.2176	Fatty acids	$[M−H]^−$
17	FA 16:3	31.80	249.1499	Fatty acids	$[M−H]^−$
18	FA 18:0	37.10	283.2643	Fatty acids	$[M−H]^−$
19	FA 18:1	35.66	281.2488	Fatty acids	$[M−H]^−$
20	FA 18:2	34.40	279.2328	Fatty acids	$[M−H]^−$
21	FA 18:3	33.30	277.2170	Fatty acids	$[M−H]^−$
22	FA 18:4	29.17	275.1995	Fatty acids	$[M−H]^−$
23	FA 20:0	31.79	311.2011	Fatty acids	$[M−H]^−$
24	FA 20:6	27.55	299.1866	Fatty acids	$[M−H]^−$

25	FA 22:5	36.50	329.2961	Fatty acids	$[M-H]^-$
26	OxFA 16:2	29.16	265.1480	Fatty acids	$[M-H]^-$
27	OxFA 18:0	31.67	297.1749	Fatty acids	$[M-H]^-$
28	OxFA 18:1	30.14	295.2281	Fatty acids	$[M-H]^-$
29	OxFA 18:2	29.21	293.2125	Fatty acids	$[M-H]^-$
30	OxFA 18:3	29.55	291.1970	Fatty acids	$[M-H]^-$
31	OxFA 18:5	25.12	287.2229	Fatty acids	$[M-H]^-$
32	Lyso PC 14:0	29.80	468.3085	Lipids	$[M+H]^+$
33	Lyso PC 16:0	30.20	496.3398	Lipids	$[M+H]^+$
34	Lyso PC 16:1	28.70	494.3243	Lipids	$[M+H]^+$
35	Lyso PC 18:0	30.80	524.3698	Lipids	$[M+H]^+$
36	Lyso PC 18:1 sn-1	29.30	522.3561	Lipids	$[M+H]^+$
37	Lyso PC 18:1 sn-2	30.80	522.3561	Lipids	$[M+H]^+$
38	Lyso PC 18:2 sn-1	28.40	520.3398	Lipids	$[M+H]^+$
39	Lyso PC 18:2 sn-2	29.30	520.3398	Lipids	$[M+H]^+$
40	Lyso PC 18:3 sn-1	28.10	518.3239	Lipids	$[M+H]^+$
41	Lyso PC 18:3 sn-2	28.30	518.3239	Lipids	$[M+H]^+$
42	Lyso PC 19:3 sn-1	28.90	532.3411	Lipids	$[M+H]^+$
43	Lyso PC 19:3 sn-2	29.10	532.3411	Lipids	$[M+H]^+$
44	Lyso PE 16:0 sn-1	29.10	454.2939	Lipids	$[M+H]^+$
45	Lyso PE 16:0 sn-2	29.30	454.2939	Lipids	$[M+H]^+$
46	Lyso PE 18:0	31.30	482.3231	Lipids	$[M+H]^+$
47	Lyso PE 18:1	29.80	480.3100	Lipids	$[M+H]^+$
48	Lyso PE 18:2sn-1	28.60	478.2925	Lipids	$[M+H]^+$
49	Lyso PE 18:2sn-2	28.70	478.2925	Lipids	$[M+H]^+$
50	Lyso PE 18:3sn-1	27.70	476.2772	Lipids	$[M+H]^+$
51	Lyso PE 18:3sn-2	27.90	476.2772	Lipids	$[M+H]^+$
52	PC 30:6 sn-1	27.40	694.4457	Lipids	$[M+H]^+$
53	PC 30:6 sn-2	27.70	694.4457	Lipids	$[M+H]^+$
54	Demethyl-vanillic acid 4-*O*-β-D-6-*O*-benzoylglucopyranoside	19.75	419.0985	others	$[M-H]^-$
55	Pinoresinol-4-*O*-glucoside	15.17	519.1873	others	$[M-H]^-$
56	Abscisic acid	19.91	263.1290	others	$[M-H]^-$
57	2-Isopropylmalic acid	5.11	175.0614	others	$[M-H]^-$
58	Galactonic acid	0.76	195.0511	others	$[M-H]^-$
59	Pantothenic acid	2.67	218.1032	others	$[M-H]^-$
60	Dichlorogelignate	5.23	705.1672	others	$[M-H]^-$
61	Glucose	0.76	179.0562	others	$[M-H]^-$
62	Citric acid	6.10	191.0186	others	$[M-H]^-$
63	Malic acid	0.90	133.0131	others	$[M-H]^-$
64	Fumaric acid	0.90	115.0025	others	$[M-H]^-$
65	Quinic acid	0.79	191.0561	others	$[M-H]^-$

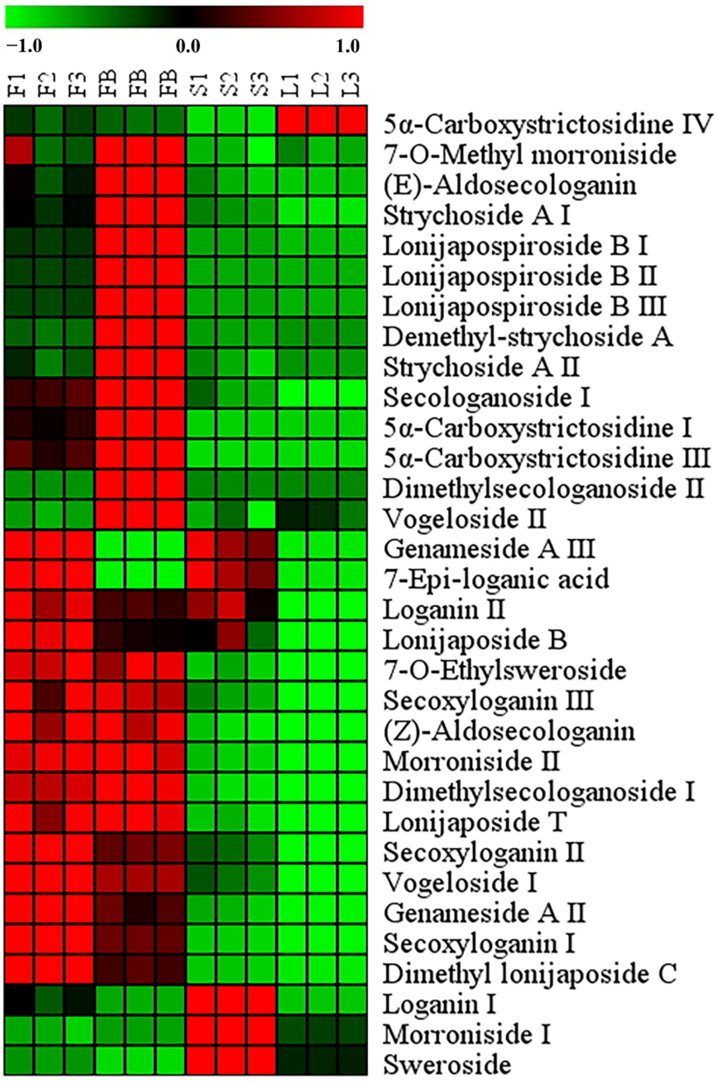

Figure S1. Heat map of differential iridoids in four different tissues of LJT.
